# RNA seq analyses of chicken reveals biological pathways involved in acclimation into different geographical locations

**DOI:** 10.1038/s41598-020-76234-8

**Published:** 2020-11-06

**Authors:** Himansu Kumar, Hyojun Choo, Asankadyr U. Iskender, Krishnamoorthy Srikanth, Hana Kim, Asankadyr T. Zhunushov, Gul Won Jang, Youngjo Lim, Ki-Duk Song, Jong-Eun Park

**Affiliations:** 1 Division of Animal Genomics and Bioinformatics, National Institute of Animal Science, RDA, Wanju, 55365 Korea; 2 Poultry Reasearch Institute, National Institute of Animal Science, RDA, Pyeonchang, 25342 Korea; 3 Institute of Biotechnology, National Academy of Science of Kyrgyzstan, Bishkek, 720071 Kyrgyzstan; 4 Department of Agricultural Convergence Technology, JeonBuk National University, Jeonju, 54896 Korea

**Keywords:** Computational biology and bioinformatics, Genetics

## Abstract

Transcriptome expression reflects genetic response in diverse conditions. In this study, RNA sequencing was utilized to profile multiple tissues such as liver, breast, caecum, and gizzard of Korean commercial chicken raised in Korea and Kyrgyzstan. We analyzed ten samples per tissue from each location to identify candidate genes which are involved in the adaptation of Korean commercial chicken to Kyrgyzstan. At false discovery rate (FDR) < 0.05 and fold change (FC) > 2, we found 315, 196, 167 and 198 genes in liver, breast, cecum, and gizzard respectively as differentially expressed between the two locations. GO enrichment analysis showed that these genes were highly enriched for cellular and metabolic processes, catalytic activity, and biological regulations. Similarly, KEGG pathways analysis indicated metabolic, PPAR signaling, FoxO, glycolysis/gluconeogenesis, biosynthesis, MAPK signaling, CAMs, citrate cycles pathways were differentially enriched. Enriched genes like *TSKU, VTG1, SGK, CDK2* etc. in these pathways might be involved in acclimation of organisms into diverse climatic conditions. The qRT-PCR result also corroborated the RNA-Seq findings with R^2^ of 0.76, 0.80, 0.81, and 0.93 for liver, breast, caecum, and gizzard respectively. Our findings can improve the understanding of environmental acclimation process in chicken.

## Introduction

Recently, consumption of chicken meat has increased worldwide because of its rich nutrition, low cost, high quality protein, low cholesterol, and low fat^1^. Climatic changes have a significant effect on development, growth, physiology, immunity and productivity of chicken^2^. Improving adaptation efficiency of the birds to different climatic condition is an important economic goal in production of Korean commercial chicken^3^. Kyrgyzstan imports significant amount of agricultural products including Korean commercial chicken (Hanhyup-3)^4^. However there are considerable differences in climatic conditions between Korea and Kyrgyzstan, which could have significant impact on Korean commercial chicken productivity in Kyrgyzstan. Diverse environmental parameters between two countries such as, Korea located at 250 m and Kyrgyzstan at 2500 m above mean sea level. The average humidity in Korea is 70% while in Kyrgyzstan it’s about 40%. Environmental stresses such as temperature, humidity, altitude, and latitude negatively affects livestock productivity^5^. Profiling transcriptome of various tissues allows interpreting the genetic response of the animal during adaptation to new climatic condition^6^. The involvement of liver in carbohydrate, protein, and lipid metabolism, bile secretion, immune defense, and various other metabolic functions are very well known^7^. Similarly breast, gizzard, and cecum are metabolically active organs^8,9^. Though these organs are central for multiple metabolic, digestive, and productive activities, little is known about their transcriptome response in regulation of molecular mechanism during adaptation to climatic conditions^10^. It is an utmost need to explore these organ’s transcriptome profile to know their response during environmental changes^11^. Transcriptome analysis has been widely applied to explore and identify differentially expressed genes (DEGs) involved in adaptation of chicken in various climatic stresses^12^. Measuring the gene expression also facilitates the pathways involved during environmental stresses^13^. The availability of the chicken reference genome sequence provides an opportunity for detailed analysis of stress resistant genes through their transcriptome^14^. Multiple reports confirm that even mild change (32–35 °C) in environmental temperature may negatively affect fertility in chicken^15–17^.

We considered four tissues including breast, liver, cecum, and gizzard of Korean commercial chicken (Hanhyup-3) raised in two diverse climatic conditions in Korea and Kyrgyzstan respectively, for transcriptome analysis through RNA-Seq. We investigated GO and KEGG pathways to identify significantly enriched GO terms and pathways involved in acclimatization into diverse environmental conditions. Our integrated study also provides tissue level insights into the molecular mechanisms involved in response to such different conditions.

## Results

Transcriptome of four tissues; liver, breast, cecum, and gizzard of ten chickens (4 tissues /bird) from each location i.e. Korea and Kyrgyzstan have been compared. High-throughput RNA-Seq using Illumina HiSeq 2500 was performed and different quality measures of raw data have been measured through Illumina package bcl2fastq such as total bases, read count, GC (%), AT (%), Q20 (%), Q30 (%). Around 67 to 108 million raw reads per sample were generated, comprising GC percentage between 45 and 48% of generated transcripts. Complete raw data statistics of both Korean and Kyrgyzstan paired end data is provided in the supplementary file 1 and 2 respectively. After trimming adapter sequences and low quality reads through reads trimming, the quality of the reads were accessed and trimmed sequences were considered for further downstream analysis.

### Mapping, differential expression, and clustering analysis of transcriptomes

**Figure 1:**
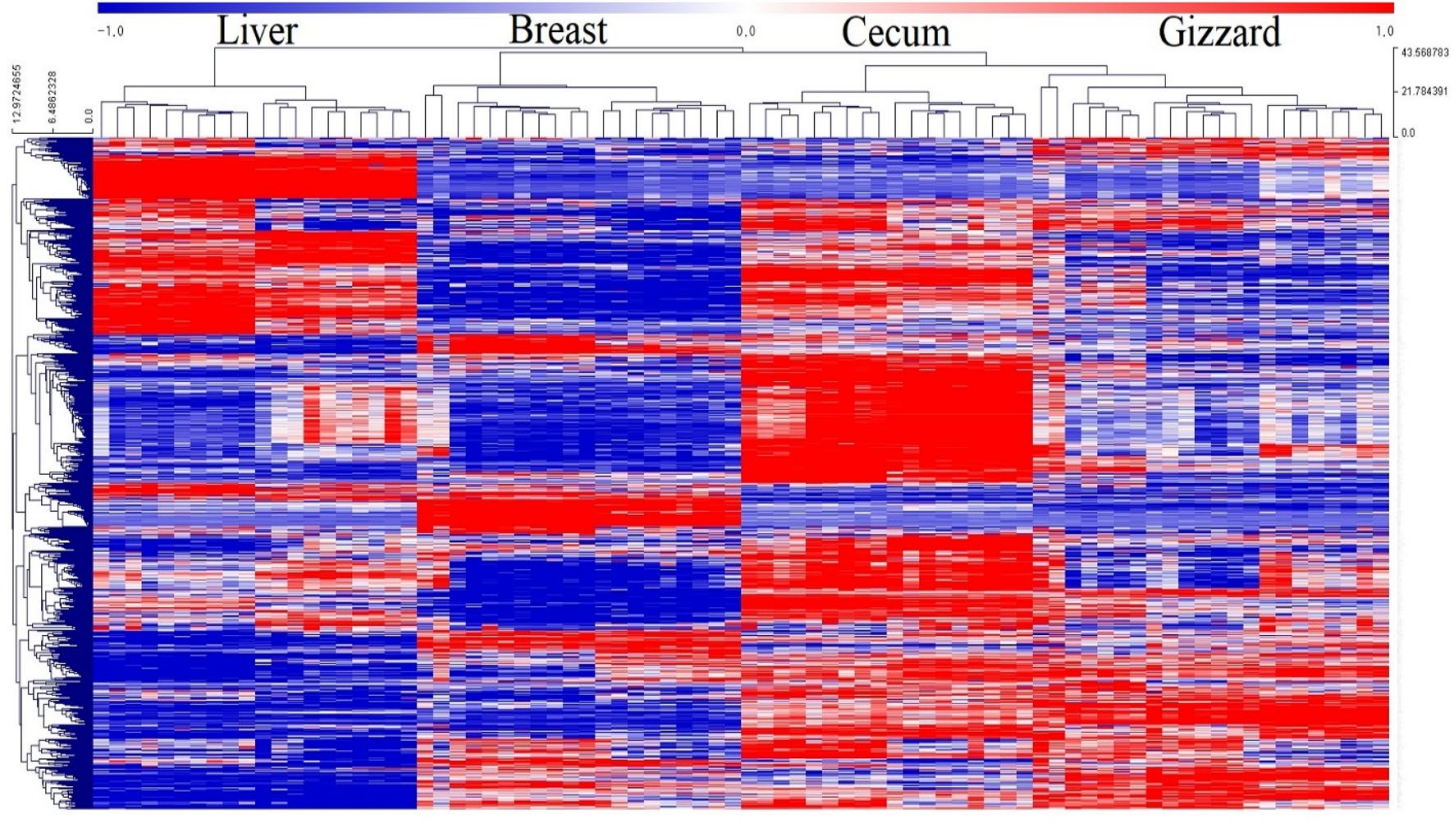
DEGs hierarchical clustering, heat map showing genes (rows) with differential expression (fold ≥ 2, FDR ≤ 0.05) among ten replicates of liver, breast, cecum, and gizzard samples, expression values are log2-transformed and median-centered by gene.

**Figure 2:**
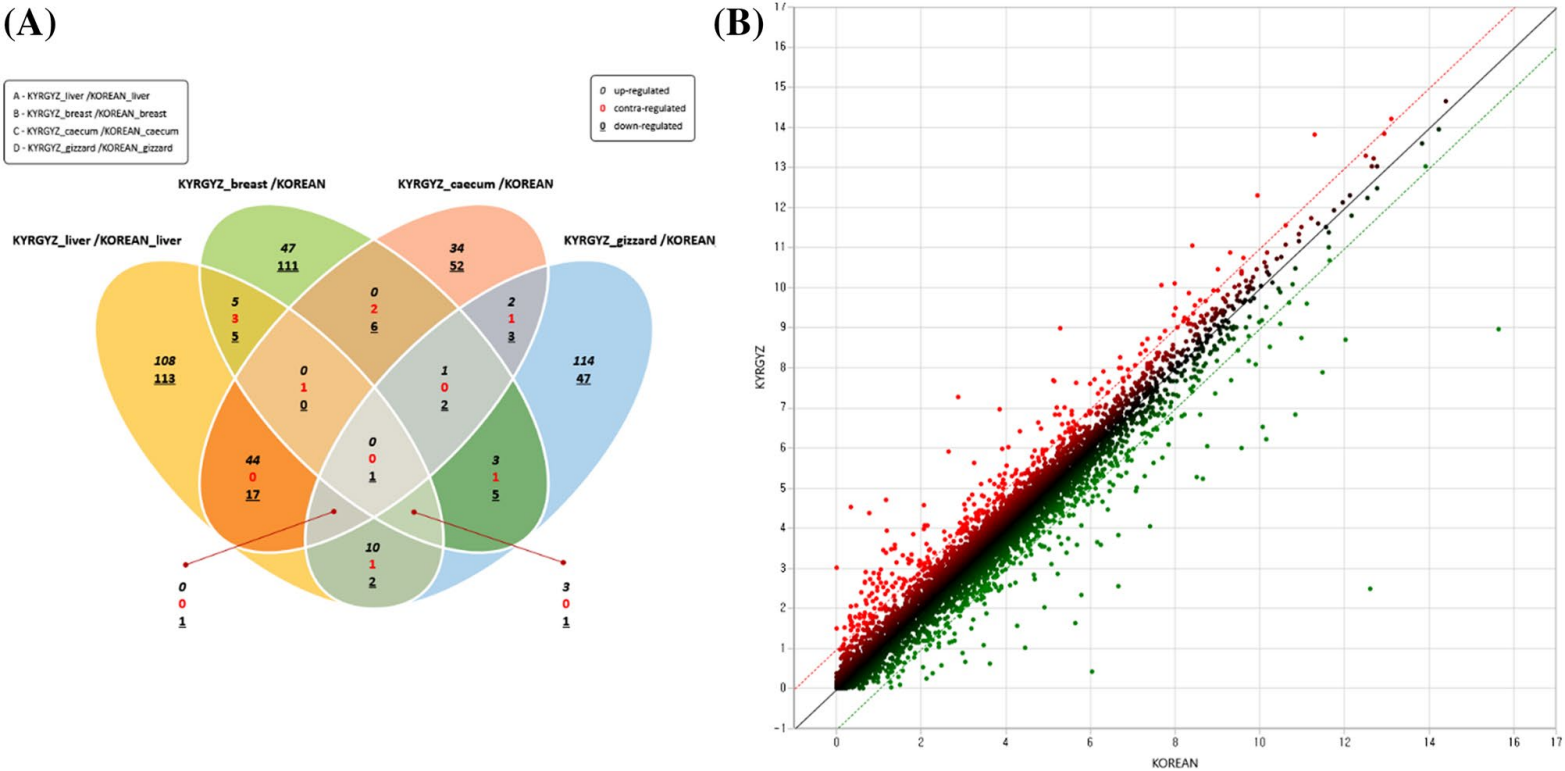
(**A**) Venn diagram of DEGs in all four tissues, (**B**) Scatter plot of DEGs identified in liver tissue between Korean and Kyrgyzstan.

Genes having low expression value (FPKM < 1) were removed and differentially expressed genes (DEGs) were identified between Korea and Kyrgyzstan chicken. According to cutoff values of fold change ≥ 2 and adjusted FDR correction p-value < 0.05, we found 174 and 141 up- and down-regulated genes respectively in liver tissue (Supplementary File 3). In case of breast tissue, 59 and 137 up- and down-regulated genes respectively (Supplementary File 4), likewise, in case of cecum 82 and 84 up- and down-regulated genes respectively (Supplementary File 5), and 135 and 62 up- and down-regulated genes respectively in case of gizzard (Supplementary File 6). We performed hierarchical clustering for all 80 samples to explore the relative amount of variation between each location and compared to that among the four tissues. A heatmap of normalized gene expression (FPKM) counts for each tissue was generated by comparing Korea and Kyrgyzstan (Fig. 1). Venn diagram and boxplot of DEGs showing up- and down-regulated genes are shown in Fig. 2A,B respectively.

### Gene ontology analysis

**Figure 3:**
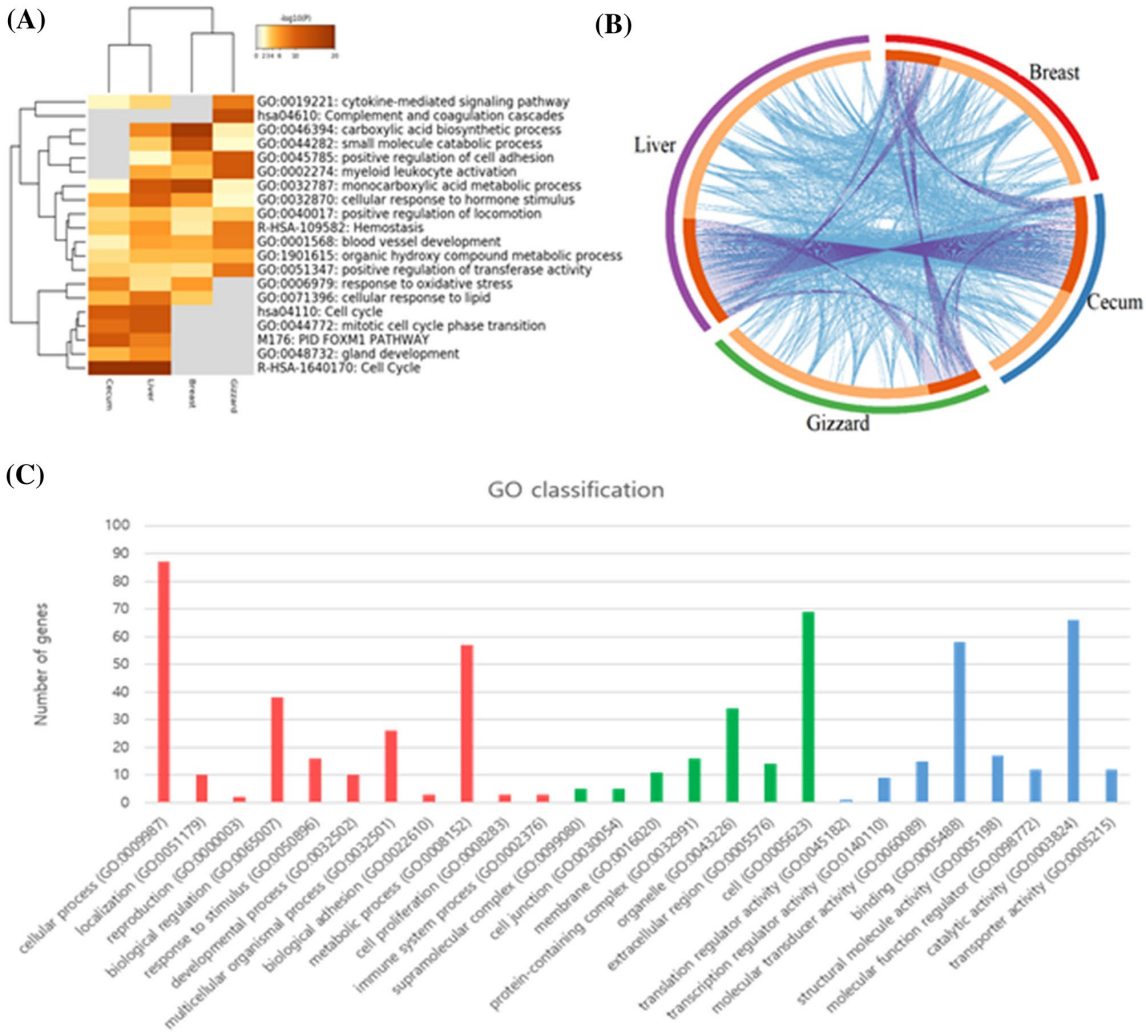
(**A**) Heatmap of enriched terms, colored by p-values (**B**) Overlap between gene lists, including the shared GO term level (**C**) GO enrichment under different environmental conditions between Korea and Kyrgyzstan. GO categories are such as biological processes, cellular components, and molecular functions are shown in red, green, and blue color respectively.

GO enriched terms in all four tissues shown in Fig. 3A^18^. A circos image has been generated to show the overlap between DE genes among four tissues (Fig. 3B), blue curved link genes shows that belong to the same enriched ontology term, and the inner circle represents gene lists, where hits are arranged along the arc. Genes that hit multiple lists are colored in dark orange, and genes unique to a list are shown in light orange (Fig. 3A).

The functional annotations and annotated genes were categorized into three groups such as molecular function, cellular component, and biological process. In case of liver, all DEGs were assigned to 27 GO terms among three major categories. Most significant (corrected p-value < 0.05) GO terms such as cellular process (GO: 0009987), cell (GO: 0005623), and binding (GO: 0005488) were enriched in biological process, cellular component, and molecular function respectively. Likewise in case of breast, cecum and gizzard, significant GO terms are metabolic and cellular process (GO: 0008152 and GO: 0009987), cell and organelle (GO: 0005623, and GO: 0043226), catalytic activity and binding (GO: 0003824 and GO: 0005488) were enriched in biological process, cellular component, and molecular function respectively (Fig. 3C).

### KEGG pathway analysis

**Figure 4:**
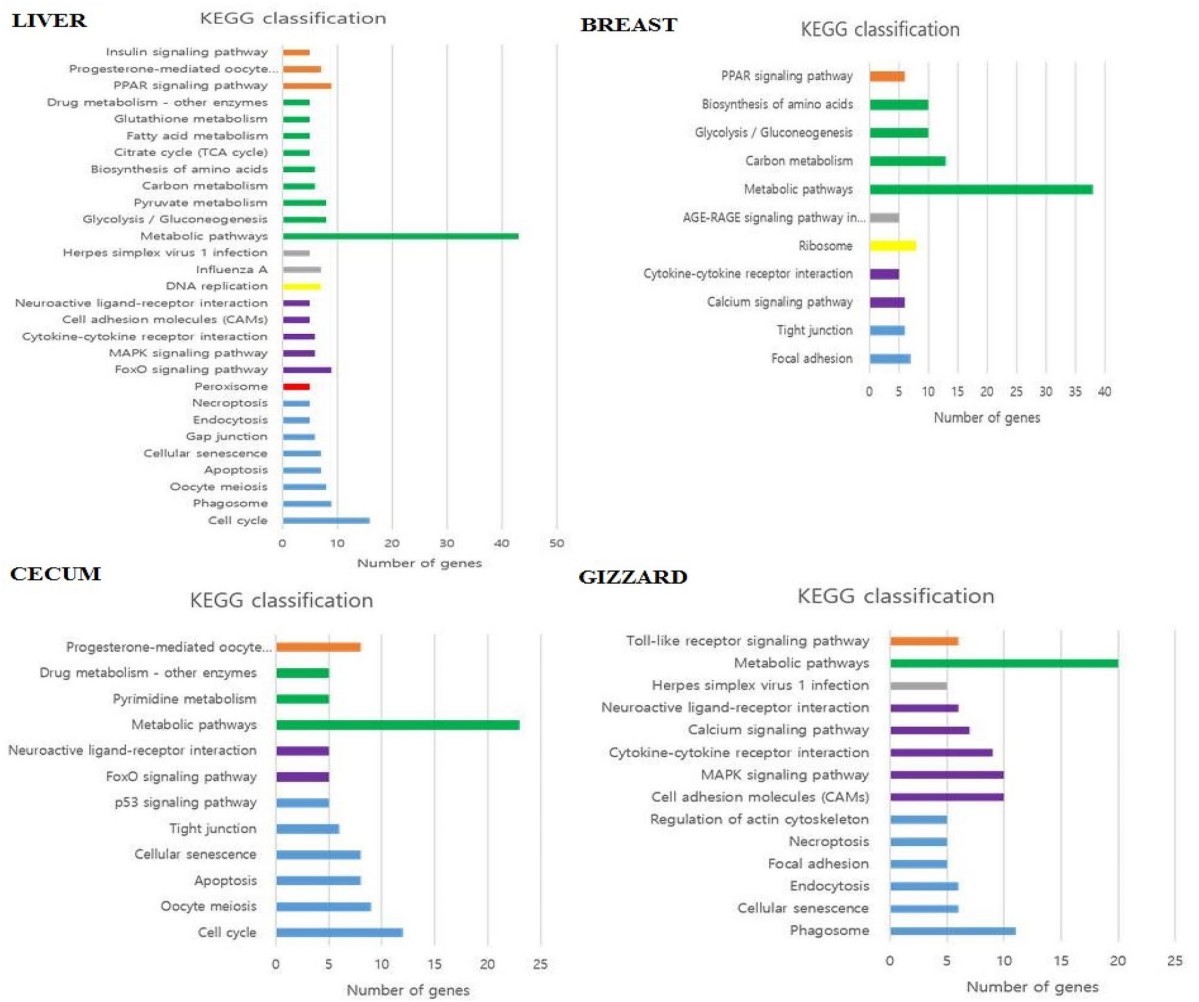
**E**nriched KEGG pathways of all four tissues such as liver, breast, caecum, and gizzard based on number of DE genes.

KEGG pathway analysis has been performed to explore the molecular interaction networks within cells and putative biological functions of DE genes. As shown in Fig. 4, metabolic pathway is the most frequently enriched pathway in all four tissues during acclimatization in Kyrgyzstan environment. PPAR signaling pathway, biosynthesis of amino acids, glycolysis/gluconeogenesis, cytokine-cytokine receptor interaction, and carbon metabolism were commonly enriched in liver and breast both tissues. Likewise, some of the pathways such as progesterone mediated oocyte, drug metabolism, neuroactive ligand receptor interaction, FoxO signaling pathway, cellular senescence, apoptosis, oocyte meiosis, cell cycle was commonly enriched in liver and cecum tissues. Similarly, pathways such as neuroactive ligand receptor interaction, neuroactive ligand receptor interaction, cell adhesion molecules, MAPK signaling pathway, necroptosis, endocytosis, cellular senescence, and phagosome were commonly enriched in liver and gizzard (Fig. 4). The DE genes involved in all pathways have been provided in supplementary materials. Ingenuity Pathway Analysis (IPA) was used to decipher the genetic networks of DE genes involved in particular pathways during acclimatization in Kyrgyz environment. As shown in Figs. 6,7,8and9, network of enriched pathways involved in tissues showing the DE genes. Color gradient blue to red indicates the down to upregulated genes respectively.

### Validation of RNA-seq results by qRT-PCR

**Figure 5:**
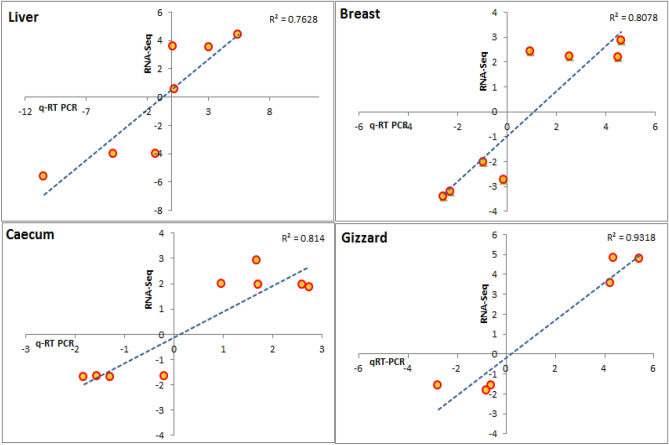
The gene expression correlation results of top 5 up and down regulated genes and the mRNA expression level obtained from real-time PCR analysis and RNA-seq genes shown as liver, breast, cecum, and gizzard.

We have validated the DE genes obtained from RNA-seq data by real-time RT–PCR analysis. The expression of top 5 up and down regulated genes was analyzed in the 10 samples of each location Korea and Kyrgyzstan. Our analysis revealed similar genetic expression pattern of all the selected genes in real-time PCR analysis we found in RNA-seq data. The statistical analysis also showed very good correspondence as correlation coefficient (R^2^) of liver, breast, caecum, and gizzard are 0.7628, 0.8078, 0.814, and 0.9318 respectively among the results of real-time PCR and RNA-Seq data analyses as shown in Fig. 5.

## Discussion

Genes expressed in cells of specific tissue’s represents important information for understanding the function of tissue and their physiology^19,20^. Our study cataloged the collection of expressed genes in four chicken tissues such as liver, breast, caecum, and gizzard, and reported significant changes among the tissues from each other. We identified the most abundant transcripts and the collection of genes showed tissue specific expression. GO enrichment and KEGG pathways analyses provided confidence that the dataset is of both location Korean and Kyrgyzstan is of high quality and useful for downstream analysis. Number of studies have been focused on the transcriptional changes on chicken, but little is known about the impact of environmental changes on commercially important Korean chicken breed^11,21^.

### Impact of changes on liver tissue between Korean and Kyrgyzstan environment

**Figure 6:**
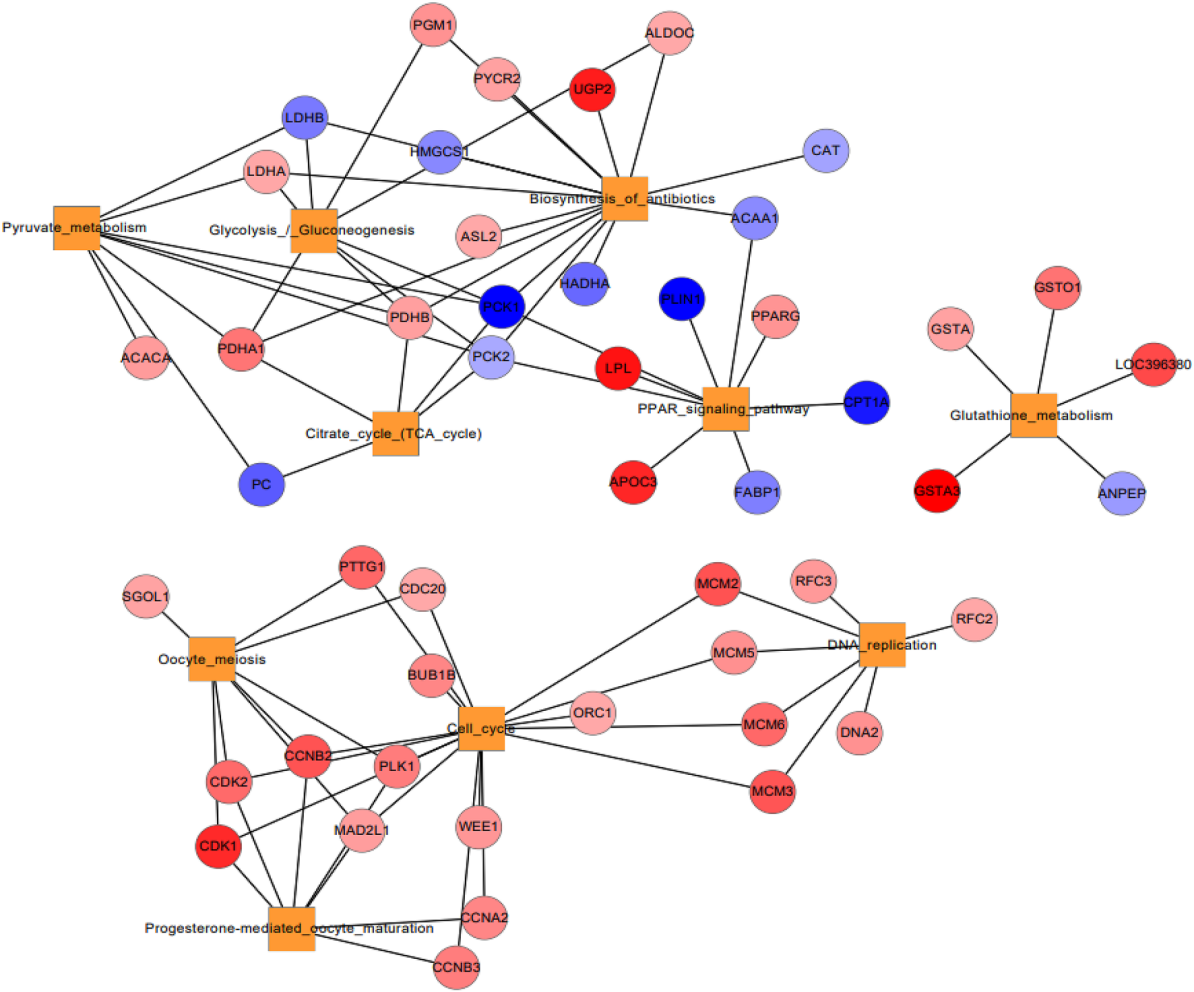
Network of enriched pathways and DE genes in liver tissue. Color gradient blue to red indicates the down to upregulated genes respectively.

Liver is an important metabolic organ that plays a critical role in lipid synthesis, degradation, and transport. However, the molecular regulatory mechanisms of lipid metabolism remain unclear in chicken^22^. In response to environmental changes, 174 and 141 genes were identified as significantly up and downregulated respectively in liver tissue. Differences in expressed genes were found between the Korean and Kyrgyzstan environment, including highly expressed novel genes and pathways^7^. Significantly important changes were observed in pyruvate metabolism related genes such as *LDHA, ACACA, PDHA1, PDHB*, are upregulated, whereas *PCK1, PCK2, LDHB* and *PC* were down-regulated (Fig. 6). Imagawa, T., et al. (2006) indicated that expression of lactate dehydrogenase (LDH) involved in inter-conversion of lactate and pyruvate^23^. *ACACA* is a significant enzyme in TCA cycle and under changed nutritional condition pyruvate is carboxylate by the pyruvate carboxylase enzyme^24^. Likewise, in cell cycle pathway (*CDK1-2, CDC20, MCM1-3, PTTG1*), Oocyte meiosis (*SGOL1, PTTG1, CDC20, CCNB2, CDK2, CDK1*), Progesterone mediated oocyte maturation (*CDK1, CDK2, CCNB2, MAD2L1, PLK1, CCNB3, CCNA2*), DNA replication (*RFC3, RFC2, DNA2, MCM-2,3,5,6*), all genes were downregulated. In PPAR signaling pathway *LPL* and *APOC3* genes were upregulated whereas *CPT1A, PLIN1, PPARG, FABP1, PCK2, PCK1* were downregulated. In Glycolysis/Gluconeogenesis upregulated genes were *PDHA1*, *PDHB, LDHA*, and downregulated genes were *LDHB, HMGCS1, and LDHBPCK1* (Fig. 6). In citrate cycle *PDHA1*, *PDHB* were upregulated and genes such as *PC*, *PCK1*, *PCL2* were downregulated. Similarly in GO terms investigations, energy related GO terms such as pyruvate, lactate, glycerol catabolic process, phosphorylation (Table 1), glutathione, glutathione transferase activity were significantly enriched in various categories of GO^25^.

**Table 1:** Tissue wise enriched KEGG pathways and associated genes.

Tissue	KEGG pathway	Count	Fold enrichment	− log10 (p-value)	Genes
Liver	Cell cycle	16	4.728913	6.076795	*CDK1, CDC20, MCM2, PTTG1, MCM3, CDK2, MCM5, WEE1, MCM6, CCNB3, MAD2L1, CCNB2, PLK1, BUB1B, CCNA2, ORC1*
	Pyruvate metabolism	8	7.649713	4.244594	*LDHB, LDHA, ACACA, PDHA1, PCK2, PDHB, PCK1, PC*
	DNA replication	7	7.585965	3.629713	*DNA2, RFC3, RFC2, MCM2, MCM3, MCM5, MCM6*
	PPAR signaling pathway	9	4.959348	3.438575	*LPL, PLIN1, APOC3, PPARG, FABP1, PCK2, CPT1A, PCK1, ACAA1*
	Glycolysis/gluconeogenesis	8	5.099808	3.099527	*LDHB, LDHA, ALDOC, PGM1, PDHA1, PCK2, PDHB, PCK1*
	Biosynthesis of antibiotics	15	2.679501	2.937769	*LDHB, LDHA, ASL2, ALDOC, HMGCS1, PCK2, HADHA, PDHB, PCK1, PYCR2, PGM1, PDHA1, CAT, UGP2, ACAA1*
	Citrate cycle (TCA cycle)	5	5.805585	2.016859	*PDHA1, PCK2, PDHB, PCK1, PC*
Breast	Biosynthesis of antibiotics	20	5.001735	8.187750758	*LDHA, PFKL, PGAM1, IDH3B, LSS, PFKM, FDFT1, GART, PKM, GOT2, GPI, NME5, TPI1, PGP, SQLE, PGM1, ENO3, SUCLA2, PGK1, GAPDH*
	Glycolysis/gluconeogenesis	11	9.817131	7.050261023	*PKM, GPI, LDHA, TPI1, PFKL, PGM1, PGAM1, ENO3, PFKM, PGK1, GAPDH*
	Carbon metabolism	14	6.637719	7.017104542	*PKM, GOT2, GPI, PGP, TPI1, PFKL, MCEE, PGAM1, ENO3, IDH3B, PFKM, PGK1, SUCLA2, GAPDH*
	Biosynthesis of amino acids	11	8.783749	6.56804503	*PKM, GOT2, GLUL, TPI1, PFKL, PGAM1, ENO3, IDH3B, PFKM, PGK1, GAPDH*
	Metabolic pathways	37	1.590259	2.732261046	*LDHA, ATP5B, PGAM1, LSS, HIBADH, FDFT1, GOT2, PKM, MTHFD2, PGP, TPI1, CRYL1, ST6GALNAC4, MCEE, CKMT2, ENO3, SUCLA2, BDH1, GAPDH, DHCR24, PDXK, GATM, PFKL, AMACR, EPHX2, IDH3B, PFKM, GART, GPI, NME5, GLUL, SQLE, GLS, PGM1, PGK1, AMY1A, MPST*
	Steroid biosynthesis	4	10.7096	2.265676219	*SQLE, LSS, FDFT1, DHCR24*
	PPAR signaling pathway	6	4.628724	2.062731216	*SLC27A1, LPL, CPT2, APOC3, ADIPOQ, FABP5*
Caecum	Cell cycle	12	6.206699	5.660312	*CDK1, CCNB3, CCNB2, MAD2L1, PLK1, BUB1, BUB1B, CDC20, MCM2, PTTG1, CCNA2, CDK2*
	Oocyte meiosis	9	5.7534	3.891816	*CDK1, CCNB2, MAD2L1, PLK1, SGOL1, BUB1, CDC20, PTTG1, CDK2*
	Progesterone-mediated oocyte maturation	8	6.068772	3.560625	*CDK1, CCNB3, CCNB2, MAD2L1, PLK1, BUB1, CCNA2, CDK2*
	p53 signaling pathway	6	5.596204	2.416468	*CDK1, CCNB3, CCNB2, CYCS, CDK2, GTSE1*
	Glutathione metabolism	4	5.98892	1.562493	*GPX2, GSTA3, RRM1, GSTO1*
	Pyrimidine metabolism	5	3.307834	1.214186	*DCTD, RRM1, DGUOK, UCK2, TK1*
Gizzard	Cell adhesion molecules (CAMs)	10	4.779485	3.730487	*PTPRC, CD86, CD80, DMA, CLDN10, ITGB2, CDH2, SDC4, YF5, DMB2*
	Phagosome	10	4.270196	3.360878	*TUBB, MBL, TFRC, C3, DMA, ITGB2, TLR4, CTSS, YF5, DMB2*
	MAPK signaling pathway	10	2.469023	1.757301	*BDNF, RAC2, MAP2K3, NTRK2, FGF13, GADD45B, FGF1, GADD45A, MYC, CACNA1B*
	Toll-like receptor signaling pathway	6	3.766004	1.701982	*CTSK, CD86, CD80, MAP2K3, TLR4, TLR7*
	Intestinal immune network for IgA production	4	6.722114	1.695366	*CD86, CD80, DMA, DMB2*
	Cytokine-cytokine receptor interaction	7	2.497772	1.241337	*CCR5, CCR8L, CCR2, LOC418666, IL2RG, XCR1, PRL*
	Calcium signaling pathway	7	2.26506	1.07805	*CCKAR, SLC8A1, ATP2A3, ERBB2, BDKRB2, CAMK2A, CACNA1B*
	Biosynthesis of amino acids	4	3.655887	1.032526	*GLUL, MAT1A, ALDOB, PAH*

### Impact of changes in breast muscle transcriptome between Korean and Kyrgyzstan environment

**Figure 7:**
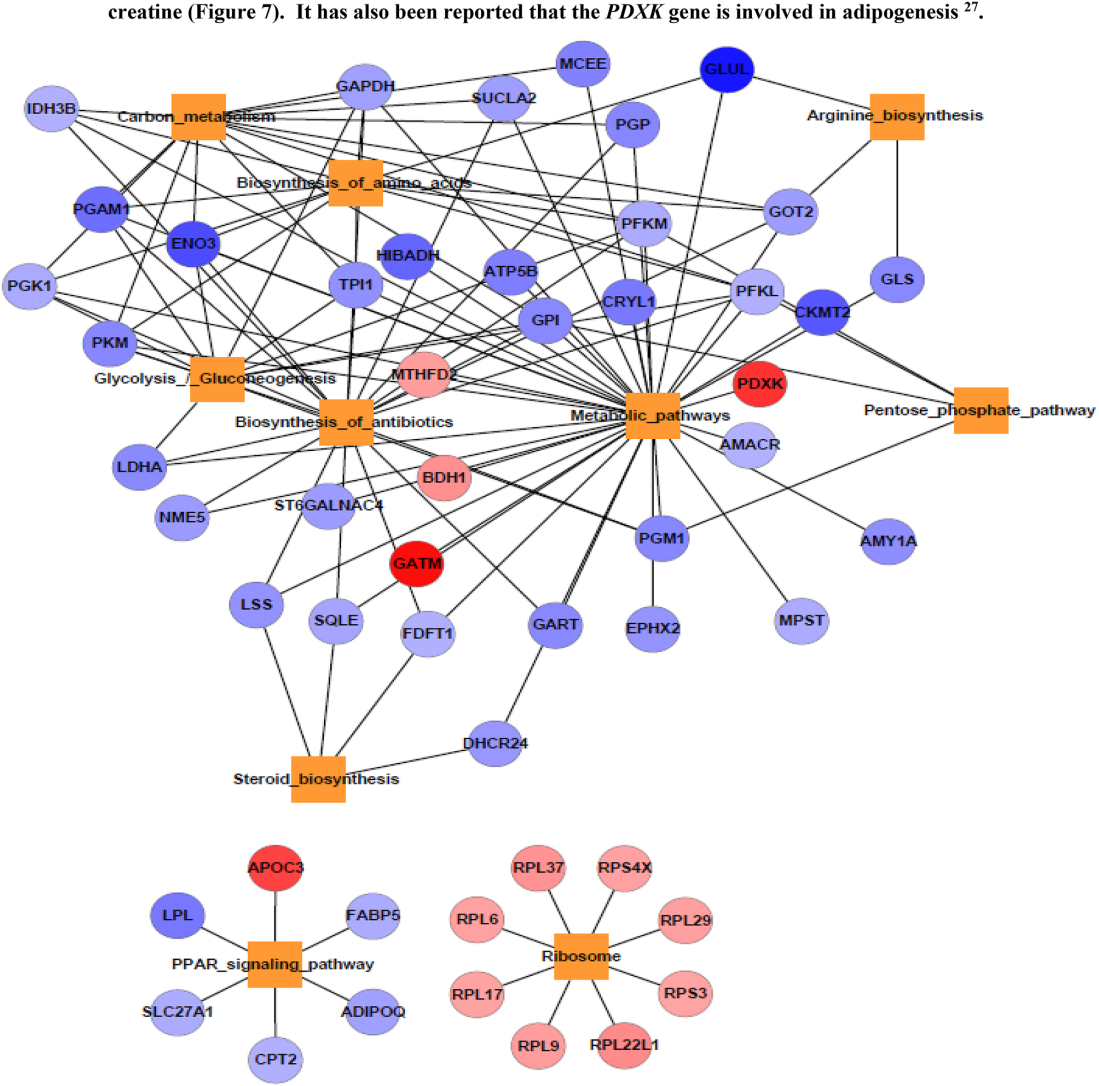
Network of enriched pathways and involved DE genes in breast muscle. Color gradient blue to red indicates the down to upregulated genes respectively.

In recent years, the importance of transcriptome changes in breast muscle function of Korean chickens and of the corresponding effects on meat quality has increased. It has been reported that Korean chickens are more sensitive to heat stress during transport and at high ambient temperatures than other chickens^26^. In response to environmental challenges, 60 and 136 genes were identified as significantly up and downregulated respectively in breast muscle. We validated top upregulated genes such as *GATM, PDXK, PIT54, SLC38A4* and top downregulated genes are *MN, NRGN, GLUL, PDK4, GATM* involved in energy metabolism in muscle tissues by catalyzing the biosynthesis of guanidinoacetate which is a precursor of creatine (Fig. 7). It has also been reported that the *PDXK* gene is involved in adipogenesis^27^.

GO terms such as glycolytic pathway (*PKM, BPI, TPI1,* and *PFKL* etc.), gluconeogenesis (*GOT2, GPI, TPI1, PGAM1, ENO3,* and *PGK1* etc.), myosin complex (*MYH1E-F, MYL1* etc.). Likewise KEGG pathways investigation showed that PPAR signaling pathway, ribosome, steroid biosynthesis, metabolic pathways, biosynthesis of antibiotics, pentose phosphate pathways, glycolysis/gluconeogenesis, carbon metabolism, pentose phosphate pathways were enriched in Kyrgyzstan. *APOC3* gene is downregulated in PPAR pathway, indicates that the level of triglycerides decreasing by inhibiting the hydrolysis of lipoprotein in plasma^28^.

### Impact of changes on caecum between Korean and Kyrgyzstan environment

The cecum is an important part of the animal digestive system. Recent transcriptome studies have been focused on digestive system of chicken, but the molecular mechanisms underlying chicken cecum metabolism remain poorly understood. To identify genes related to cecal metabolism and to reveal molecular regulation mechanisms, we sequenced and compared the transcriptomes of cecum. In response to environmental challenges, a total of 167 genes were identified as significantly differentiated in caecum tissue.

**Figure 8:**
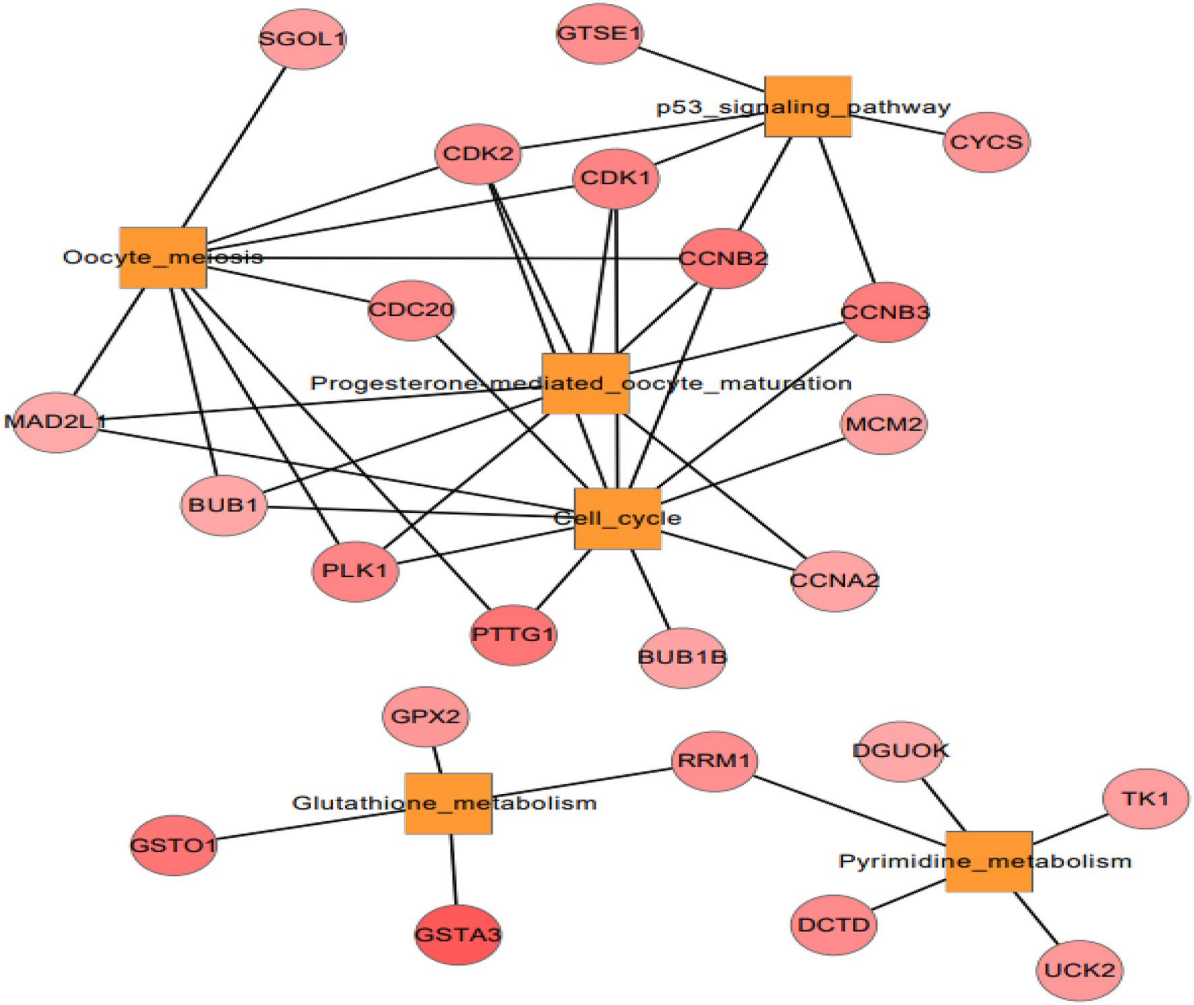
Network of enriched pathways and involved DE genes in caecum tissue.

KEGG pathways investigation indicates that Oocyte meiosis (*SGOL1, MAD2L1, BUB1, PLK1, CDC20, CDK2*), p53-signaling pathway (*CDK1, CDK2, CCNB2, CCNB3, CYCS, GTSE1*), progesterone mediated oocyte maturation (*CCNB2-3, CDK1-2, BUB1, MAD2L1*), cell cycle, glutathione metabolism (*PTTG1, BUB1B, CCNA2, MCM2, CCNB2-3, PLK1*), pyrimidine metabolism (*UCK2, DCTD, TK1, DGUOK, RRM1*). All mentioned genes were downregulated as shown in Fig. 8.

### Impact of changes on gizzard between Korean and Kyrgyzstan environment

Analysis of the RNA-seq data of gizzard tissue showed a total of 198 differentially expressed transcripts between the Korean and Kyrgyzstan environment. GO terms such as immune response (*CCL1, FYB, C7, C8B, CCR5* etc.), extracellular region (*CCL1, BMP4, MBL, C3, FETUB* etc.), extracellular space (*GC, BMP4, XDH* etc.) were significantly enriched in these two geographically diverse locations.

**Figure 9:**
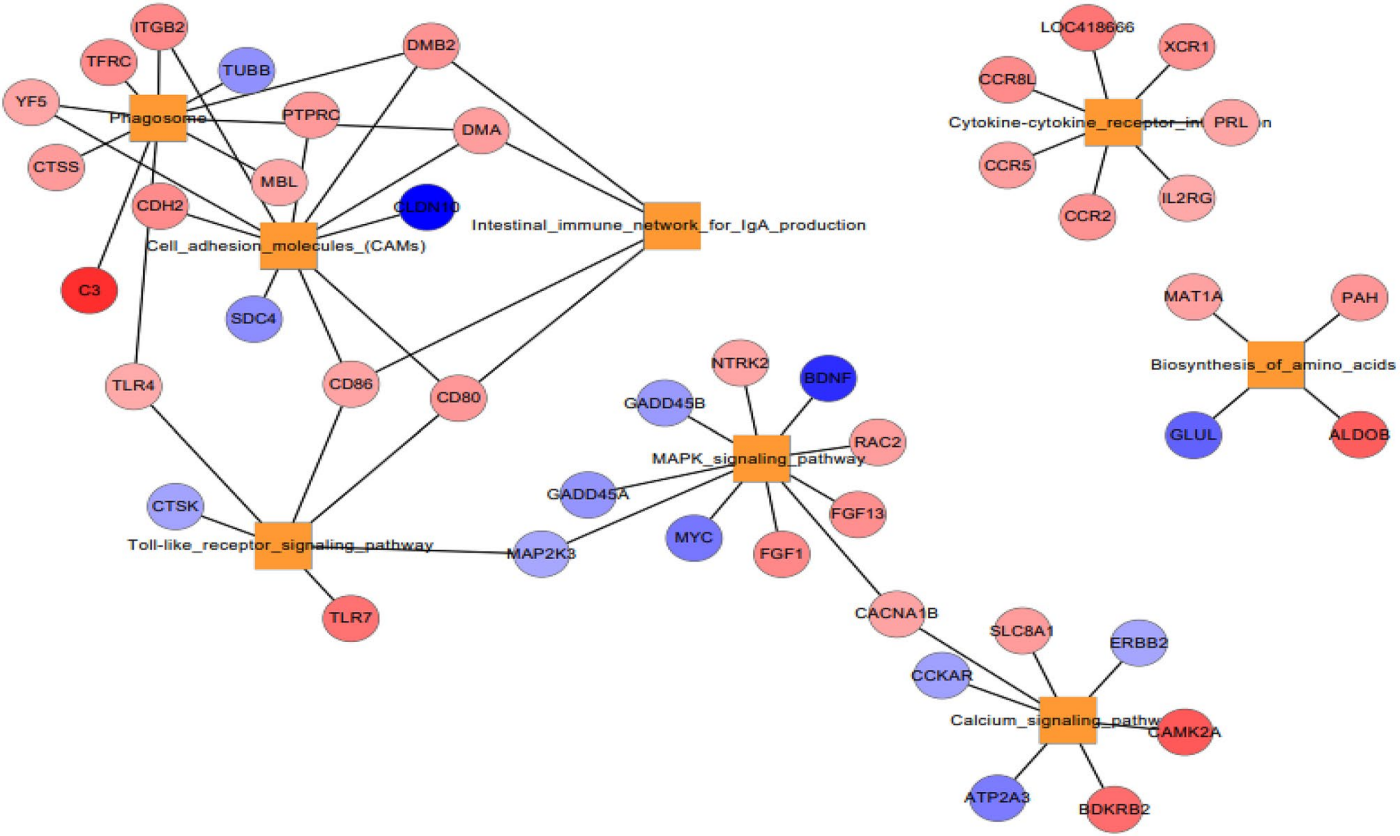
Network of enriched pathways and involved DE genes in gizzard. Color gradient blue to red indicates the down to upregulated genes respectively.

KEGG pathways showed that MAPK signaling pathway (up regulated—*NTRK2, FGF1, FGF13, RAC2, CACNA1B*, and downregulated—*MYC, MAP2K3, GADD45A, BDNF*), calcium signaling pathway (up regulated—*CAMK2A, BDKRB2, SLC8A1,* and downregulated—*ATP2A3, CDKAR, ERBB2*), toll like receptor signaling pathway (upregulated—*TLR7, CD80, CD86,* and downregulated—*CTSK*), cell adhesion molecules, phagosome, intestinal immune network for IgA production (Fig. 9).

## Conclusion

Primary objective of this study was to explore the molecular mechanism involved in tissues such as liver, breast, cecum, and gizzard when chicken encounters the environmental changes. In conclusion, our results give new insight to understand the changes in transcriptome that may occur during acclimatization into different locations. GO terms analyses showed that the metabolic and cellular process, cell and organelle, catalytic activity and binding were significantly enriched in almost all tissues during acclimatization into Kyrgyzstan. Similarly, KEGG pathway investigation showed that pathways such as PPAR, MAPK, FoxO, and CAM are significantly enriched in Kyrgyzstan environment. Our findings can be a useful resource for the transcriptomic investigations of adaptation efficiency-related genes in chicken and may provide significant clue for understanding the molecular genetic mechanisms in other chicken breeds.

## Materials and methods

### Preparation of tissues

We considered 2 months old female Hanhyup-3 chickens obtained from Hanhyup breeder, a poultry breeding company in South Korea, and grown in rearing facility of NIAS South Korea at 28 °C, humidity 80%, and pressure 1008 mbar. Four tissues such as liver, breast, cecum, and gizzard were considered for sampling. Similarly, same breeds of chicken were grown in rearing facility of Institute of Biotechnology, National Academy of Science of Kyrgyz Republic at temperature 26 °C, humidity 40%, and pressure 1003 mbar. 10 samples for each tissue from each location were considered for the experiment. Dissected tissues samples were stored at − 80 °C for further use.

### RNA isolation and quality assessment

The total RNA was extracted by using Trizol/chloroform/isopropanol method (InvitrogenTM, Carlsbad, CA, USA) from all four samples liver, breast, cecum, and gizzard. The purity and integrity of RNA was estimated by the nano photometer (IMPLEN, CA, USA). RNA quality was checked by Bioanalyzer 2100 system using RNA Nano 6000 Assay kit (Agilent Technologies, CA, USA).

### cDNA library construction, sequencing, and quality control

The Illumina TruSeq stranded library preparation kit (TruSeq Stranded Total RNA LT Sample Prep Kit) was used to prepare the library for 10 samples from each tissues and each location Korea and Kyrgyzstan. Illumina Hiseq 2500 platform was used and 100‐bp paired‐end reads were generated. The PCR products have been purified through AMPure XP system (Beckman Coulter, Beverly, USA), and Agilent Bioanalyzer 2100 system (Agilent Technologies, CA, USA) was used to check the quality of library^29^. Quality of Illumina reads were checked through FastQC software by considering Q > 30, GC-content, sequence duplication level, and other default FastQC parameters^30^. Trimmomatic software (Version 0.39) with using parameters (*ILLUMINACLIP:TruSeq3-PE.fa:2:30:10 LEADING:3 TRAILING:3 SLIDINGWINDOW:4:15 MINLEN:36) *to remove adaptor and low quality sequence and transformed it into clean and ready to use for mapping^31^.

### Reads mapping, quantification of gene expression level, and differential expression analysis

Qualified reads were mapped through Tophat2 (v2.1.1) software with default parameters to the chicken reference genome (GalGal5)^32–34^. The mapped BAM file has been used to estimate the gene expression through fragments per kilo base of transcript per million fragments mapped (FPKM) through cufflinks (version 2.2.1)^35^. Differential expression (DE) analysis of Korean and Kyrgyzstan groups (10 biological replicates from each location) was performed using the DESeq2 R package (version: 1.28.1). The P values adjustment had been done using the Benjamini and Hochberg's approach for controlling the FDR. Genes with a P value < 0.05 and fold change ≥ 2 were used as threshold for DE by DESeq2^36^.

### GO and KEGG enrichment analysis of differentially expressed genes

GO and KEGG pathways enrichment analysis were done by Database for Annotation, Visualization and Integrated Discovery (DAVID, https://david.ncifcrf.gov/)^37^ and Meatascape (https://metascape.org/gp/index.html#/main/step1)^18^. For significant enrichment, we consider Benjamini-corrected value P ≤ 0.05^38^.

### Quantitative reverse-transcription-PCR (qRT-PCR)

We carried qRT-PCR for top ten DE genes obtained from RNA-seq for the purpose of validation. Primers were designed via Primer3 (https://bioinfo.ut.ee/primer3-0.4.0/primer3/input.htm)^39^. The mRNA levels of the DEGs were normalized by the housekeeping genes β-Actin and GAPDH (Glyceraldehyde 3-phosphate dehydrogenase). The relative gene expression values were calculated using the 2 − ΔΔCt method. Finally, the correlations between RNA-Seq of top 5 up and down regulated genes and the mRNA expression level from qRT-PCR were estimated.

### Ethics statement

All experiments were performed in accordance with relevant guidelines and regulations. The experiments were also conducted according to norms approved by the National Institute of Animal Science (NIAS), South Korea. The animals used for these experiments performed on the basis of approved by ethical committee of NIAS, South Korea (Approval number: 2018-262).

## Supplementary information

Supplementary Information.

Supplementary Information.

Supplementary Information.

Supplementary Information.

Supplementary Information.

Supplementary Information.

## Data Availability

Sequencing reads are submitted into NCBI under SRA submission portal with Project Code PRJNA577590.
